# *Indy* gene variation in natural populations confers fitness advantage and life span extension through transposon insertion

**DOI:** 10.18632/aging.100634

**Published:** 2014-01-31

**Authors:** Chen-Tseh Zhu, Chengyi Chang, Robert A. Reenan, Stephen L. Helfand

**Affiliations:** Department of Molecular Biology, Cell Biology and Biochemistry, Division of Biology and Medicine, Brown University, Providence, RI 02912

**Keywords:** Drosophila melanogaster, Indy, Hoppel, natural transposon, fitness, fertility, life span, longevity, aging, selective sweep, metabolism, genetic variants, gene regulatory networks

## Abstract

Natural selection acts to maximize reproductive fitness. However, antagonism between life span and reproductive success frequently poses a dilemma pitting the cost of fecundity against longevity. Here, we show that natural populations of *Drosophila melanogaster* harbor a *Hoppel* transposon insertion variant in the longevity gene *Indy* (*I'm not dead yet*), which confers both increased reproduction and longevity through metabolic changes. Heterozygosity for this natural long-lived variant has been maintained in isolates despite long-term inbreeding under laboratory conditions and advantageously confers increased fecundity. DNA sequences of variant chromosome isolates show evidence of selective sweep acting on the advantageous allele, suggesting that natural selection acts to maintain this variant. The transposon insertion also regulates *Indy* expression level, which has experimentally been shown to affect life span and fecundity. Thus, in the wild, evolution reaffirms that the mechanism of heterozygote advantage has acted upon the *Indy* gene to assure increased reproductive fitness and, coincidentally, longer life span through regulatory transposon mutagenesis.

## INTRODUCTION

The genetic basis of longevity in species ranging from yeast to mammals has been investigated primarily through experimental alteration of expression in individual genes. Fewer studies have investigated the genetic architecture of longevity in natural populations at the level of molecular function [1]. One gene whose role in life span has been extensively studied is the *Indy* gene, in both *Drosophila* and *C. elegans* systems [2-6]. Importantly, *Indy*'s positive role in regulating metabolism and insulin sensitivity has recently been genetically demonstrated in mammals [7]. The *INDY* protein has been shown to possess dicarboxylate transporter activity [7, 8]. As such, it has been proposed that *Indy* mutations act to regulate the levels of tricarboxylic acid cycle (TCA) intermediates (e.g. citrate, succinate) via effects on transport, effectively inducing a genetic form of caloric restriction (CR) [4, 6], an intervention known to extend life span. While there is abundant evidence for the function of *INDY*, the mechanism through which experimentally-induced mutations in *Indy* increase life span have not been elucidated. Upon investigation, we found that *Drosophila* isolates from around the world vary with respect to a naturally occurring polymorphism caused by the insertion of a transposable element, *Hoppel*. This mobile element has inserted into the first intron of the *Indy* gene, and we show that it is often maintained in a heterozygous state. The presence of the *Hoppel* element in *Indy* is associated with an increase in fertility and increased longevity. We provide evidence for a selective sweep in the region where *Hoppel* has inserted, a molecular signature of recent positive selection acting on DNA sequences at the population level.

## RESULTS

### *Hoppel* polymorphism is found in *Drosophila* isolates from around the world

**Figure 1:**
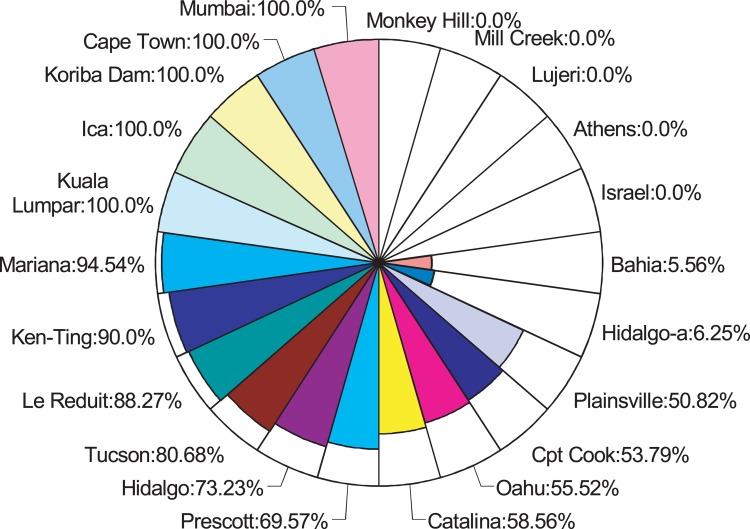
World-wide heterozygosity of *Hoppel* insertion in *Indy* Frequency of *Hoppel* insertion in isofemale inbred lines. Each part of the pie chart represents a line from a different geographic origin. The colored area in each represents the frequency of *Hoppel* + allele in that line (Table S1).

In the course of our molecular analyses of *Indy* gene structure in different *Drosophila* isolates from around the globe, we found a natural polymorphism present in numerous independent populations. This variant comprises the presence or absence of the transposable element, *Hoppel*, which has been implicated in regulating gene expression through effects on local chromatin structure [9, 10]. We sought to examine whether heterozygosity for the *Hoppel* insertion in *Indy* is maintained in other wild strains. *Hoppel* is an intronless, defective mobile element related to the *P* element, is about 1.2kb in average length, and is estimated to have 105 insertions in *D. melanogaster* euchromatin [11]. Using a PCR based screen, we found that the *Hoppel* insertion is polymorphic among 22 natural isolate lines (obtained from the *Drosophila* Species Stock Center, Table S1). Strikingly, many lines were not fixed for the insertion or wild-type alleles (12 of 22 examined lines), even after 5-50 years of inbreeding (60-600 generations) and still maintained the insertional allele at between 6-95 % frequency (Fig. 1). Located in the chromosomal map 75E region, the *Indy* gene is far from any common natural chromosomal inversions [12]. Thus, heterozygosity for this natural *Indy* variant is not likely a result of genetic hitchhiking.

### *Hoppel* polymorphisms are associated with increased fertility

**Figure 2:**
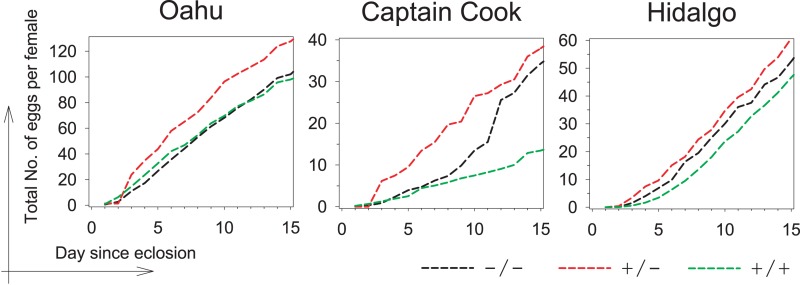
*Hoppel* insertion in *Indy* is associated with a reproductive advantage Accumulative number of eggs laid per female for each of the three *Hoppel* genotypes representing genomes with either no copies of *Hoppel* in *Indy* (−/−; in black), one copy of *Hoppel* in *Indy* (+/−; in red) or two copies of *Hoppel* in *Indy* (+/+; in green), isolated from three different populations collected from distant places or times from the wild (Oahu, Hawaii 1955, Captain Cook, Hawaii 2007 and Hidalgo, Mexico 2005).

Heterozygosity for *P*{lacW} transgene insertion at the *Indy* locus has been shown to result in life span extension without a decrement in fecundity under normal laboratory culture conditions, but with a distinct cost under reduced caloric intake [13]. We isolated *Hoppel* insertion homozygotes (referred to as +/+) and non-insertion homozygotes (referred to as −/−) from three natural lines which demonstrated heterozygosity: Oahu, Hawaii collected in 1955; Captain Cook, Hawaii collected in 2007; and Hidalgo, Mexico collected in 2005. Congenic heterozygotes (−/+) were produced by intercrossing these homozygous genotypes isolated from each natural line (Fig. S1). To examine effects on reproductive fitness, we determined the fecundity of heterozygotes and homozygotes. In all cases, we found that a single copy of the *Indy Hoppel* insertion conferred highest fecundity (Fig. 2). The strong selection for maximized fecundity in wild populations favors the preservation of the insertion allele, conferring a large benefit (~10% increased egg production) on heterozygotes over the first 15 days of reproduction. Thus, we suggest that the *Indy* gene has been the subject of selection by the process of heterosis, or heterozygote advantage.

### *Hoppel* is associated with selective sweep

**Figure 3:**
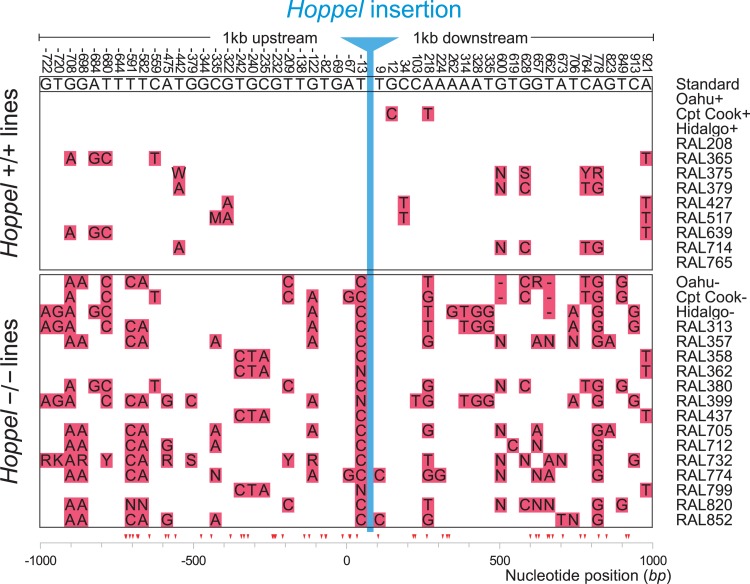
*Hoppel* insertion in *Indy* shows evidence for positive selection at the sequence level The DNA sequence polymorphisms 1000bp upstream and downstream from the *Hoppel* insertion site are shown in red blocks. The standard reference *D. melanogaster* genome sequence is at the top with nucleotide position for each polymorphic site referenced to the *Hoppel* insertion site as position 0. The upper half of the figure are strains containing the *Hoppel* insertion (Hoppel +/+) and the bottom half lines are strains that do not have the *Hoppel* insertion (*Hoppel* −/−) (Table. S1).

A molecular signature of recent positive selection acting on DNA sequences at the population level is decreased polymorphism linked to the advantageous allele, known as selective sweep [14]. Just such a selection on *Hoppel* is reflected by the DNA sequence polymorphism pattern in the region surrounding the *Hoppel* insertion site (Fig 3). We compared the sequences of *Hoppel* homozygotes +/+ and −/− isolates from the Oahu, Captain Cook and Hidalgo strains as well as the fully sequenced *Drosophila* Genomic Reference Panel (DGRP) lines (n=23) [15] which we also found were polymorphic for *Hoppel* insertions in *Indy*. Among the lines carrying *Hoppel* insertions (n=9), we found a dearth of polymorphisms in sequences neighboring the insertion site as compared to many more polymorphisms seen for sequences in the same *Indy* region in lines lacking the *Hoppel* insertion (n=14). Chromosomes from Oahu, Cpt. Cook and Hidalgo with a *Hoppel* insertion had only 2 polymorphisms over the combined 2 kb region upstream and downstream of the Hoppel insertion, while chromosomes of the same strains from Oahu, Cpt Cook and Hidalgo lacking the *Hoppel* insertion had a total of 31 polymorphisms over the same interval. For the *Drosophila* Genomic Reference Panel (DGRP) lines, or RAL lines, chromosomes containing *Hoppel* had on average 3.1-3.4 polymorphisms over the combined 2 kb region upstream and down stream of the *Hoppel* insertion site compared to chromosomes without *Hoppel* which had on average 10-11.5 polymorphisms over the same region. Since the three inbred lines, Oahu, Cpt Cook and Hidalgo are geographically and temporally separated from each other as well as from the DGRP lines, the common of reduced polymorphism around the *Hoppel* insertion can be best explained as a selective sweep encompassing the *Indy* locus due to positive selection associated with *Hoppel* rather than identity-by-descent. (Fig. 3).

### *Hoppel* is associated with increased life span

**Figure 4:**
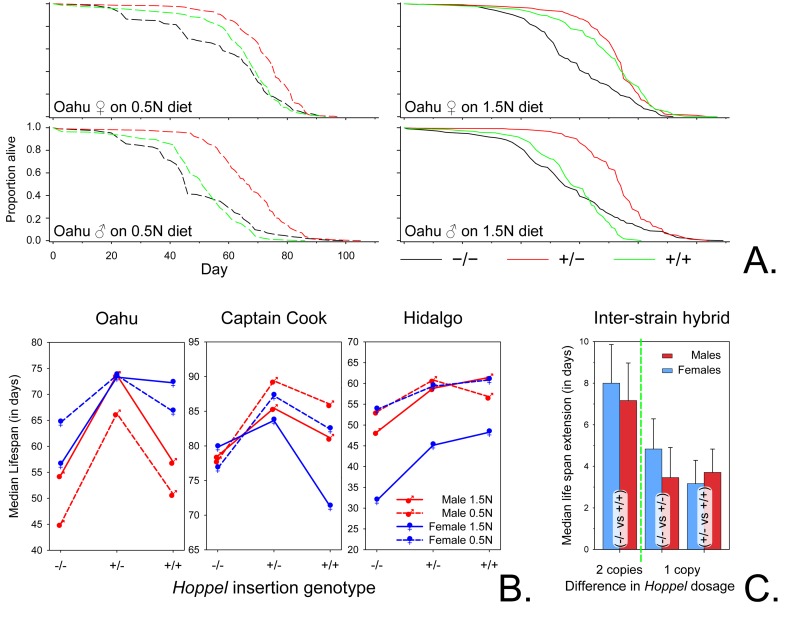
*Hoppel* insertion extends life span (**A**) Representative survivorship plots for female and male *Hoppel* genotypes isolated from Oahu lines with either no chromosome containing *Hoppel* in *Indy* (−/−; in black); one chromosome containing *Hoppel* in *Indy* (+/−; in red) or both chromosomes containing *Hoppel* in *Indy* (+/+; in green) grown on two different foods (1.5N and 0.5N diet) [29]. Survivorship plots for Captain Cook and Hidalgo lines are in Fig. S2 and tests for survivorship and maximum life span are in Table S2. (**B**) Reaction norms of median life span for Male (red) and Female (blue) Oahu, Captain Cook and Hidalgo lines for each of three different *Indy* genotypes (no chromosome containing *Hoppel* in *Indy* −/−; one chromosome containing *Hoppel* in *Indy* +/− or both chromosomes containing *Hoppel* in *Indy* +/+) grown on two different types of food (1.5N and 0.5N diet) [29]. (**C**) The effect of the dosage of each *Hoppel* containing *Indy* chromosome on life span extension for inter-strain hybrids between the Oahu, Captain Cook and Hidalgo strains represented as the mean of median life span extension in days for Males (red) and Females (blue) flies. Left of the green dotted line is the mean of the median life span extension for inter-strain hybrids in which both chromosomes have a *Hoppel* element in *Indy* (+/+) as compared to inter-strain hybrids with no *Hoppel* in *Indy* (−/−). Right of the green dotted line are shown the increase in mean of the median life span upon addition of each chromosome containing *Hoppel* in *Indy*; inter-strain hybrids with both chromosomes having a *Hoppel* in *Indy* (+/+) as compared to one chromosome having a *Hoppel* in *Indy* (+/−) and inter-strain hybrids with one chromosome having a *Hoppel* in *Indy* (+/−) as compared to inter-strain hybrids with no chromosome having a *Hoppel* in *Indy* (−/−) (Table S3). These data represent 96 separate inter-strain hybrid life spans including two different types of food (1.5N and 0.5N diet) [29]. Error bars are S.E.M.

Based upon evolutionary hypotheses regarding the interaction between reproduction and longevity, we anticipated that animals heterozygous for the insertion allele might have shorter life spans due to their increased cost in resources associated with early life reproductive success. Remarkably, when we determined the influence of the presence of the *Hoppel* insertion on life spans, we found that under two very different dietary conditions; a high calorie diet commonly used in the laboratory, and a low calorie diet likely more realistic of food availability in the wild, heterozygote animals (−/+) for *Hoppel* significantly outlive congenic homozygotes lacking the *Hoppel* insertion in *Indy* (−/−) (Figs. 4A, 4B) despite having a higher early reproductive output (Fig. 2). While the reproductive advantage of being heterozygous for the *Hoppel* variant of *Indy* (−/+) is the likely force maintaining heterozygosity, these data demonstrate a naturally occurring genetic variant that, in keeping with similar previous experimental data, is capable of extending life span [4].

To demonstrate that the life span extension is likely due to the insertion of *Hoppel* at the *Indy* locus (versus other genetic background effects within each strain), we examined the life span of inter-line hybrids where −/− and +/+ from each of the three natural isolate lines were crossed to their counterparts from another line. In these inter-line hybrids, with −/−, +/+ and −/+ genotypes at the *Indy* locus (and the rest of the genome being heterozygous at all loci), we continued to find a strong association between the presence of *Hoppel* in *Indy* and life span extension (Fig. 4C). Examination of the results of 96 separate life spans from these inter-line crosses indicates that on average, a one dose increase of *Hoppel* insertion extends median life span by ~5 days, while a two dose increase extends median life span by ~8 days.

### *Hoppel* affects *Indy* mRNA expression

**Figure 5:**
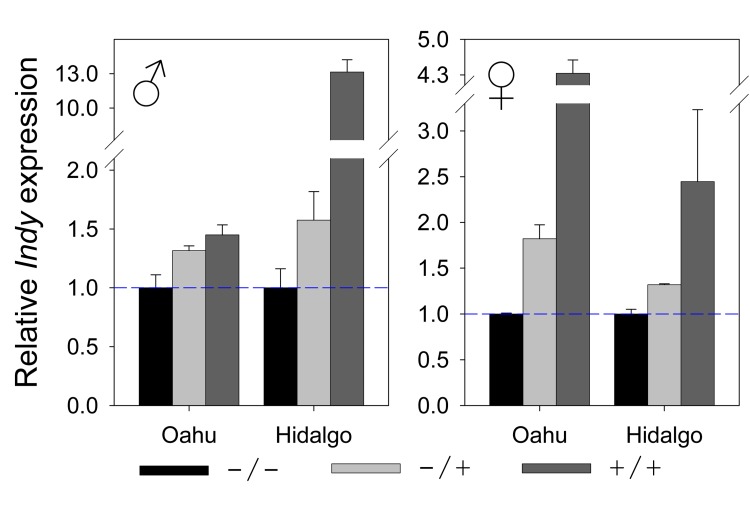
*Hoppel* insertion is positively correlated with *Indy* expression levels *Indy* mRNA expression for female and male *Hoppel* genotypes isolated from Oahu and Hidalgo lines measured by qPCR: with either no chromosome containing *Hoppel* in *Indy* (−/−; in black); one chromosome containing *Hoppel* in *Indy* (+/−; in light gray) or both chromosomes containing *Hoppel* in *Indy* (+/+; in dark gray). Relative expression level of *Indy* is compared to the expression level for the strains having no chromosome containing *Hoppel* in *Indy* (−/−). Error bars are S.E.M

Examination of the molecular underpinnings of how *Hoppel* insertion at *Indy* affects longevity suggested that *Hoppel*, which is inserted within the 1st intron of *Indy*, and is 2216 base pairs upstream of the translational start, may be exerting its action by modulating the level of *Indy* expression. Therefore, we performed mRNA expression studies of *Indy* in various allelic combinations. The expression level of *Indy* was positively correlated with increasing *Hoppel* insertion dosage: the heterozygote being intermediate (Fig. 5). Heterozygosity for the *Hoppel* insertion was shown to confer the longest life span in 10 out of the 12 life spans (Fig. 4B) in males and females, independent of caloric intake. Interestingly, the effect of the *Hoppel* insertion in these wild strains on *Indy* transcription is reminiscent of the effect of the experimentally induced mutation, *P*{lacW}*Indy^206^*, which is a transposon insertion into the *Hoppel* in *Indy* residing in the normal laboratory strain that also modulates *Indy* transcription [4, 6].

## DISCUSSION

The role of the *Indy* gene in longevity, based upon molecular genetic interventions in invertebrates, has been controversial [2-6]. Here, we show that natural selection has maintained standing genetic variation at the *Indy* locus in the wild, with phenotypic consequences, demonstrating that this gene has an evolved role in metabolism, fecundity, and longevity determination. Standard evolutionary paradigms that emphasize metabolic tradeoffs invoke an inverse relationship between early life fecundity and longevity. For instance, when populations are selected for late-life fecundity, they are associated with increased life span [16-19]. However, a recent study suggests that the inverse correlation between lifespan and fecundity may not be a conserved feature of the genetic architecture of longevity [20]. We show, that at least in this case, the molecular lesion caused by *Indy Hoppel* insertion uncouples this association, allowing for early reproductive success (high fitness) as well as increased longevity. Nevertheless, homozygosity at *Indy* (in particular the insertion variant), results in decreased fitness, thereby favoring the maintenance of the insertion allele in the heterozygous state, providing one of the few examples of a natural variant transposable element insertion conferring adaptive value in a organism.

The molecular mechanism, by which the heterozygote advantage of *Indy* on fecundity and longevity is mediated, in both variants in the wild and through experimental manipulations in the laboratory [4, 6], involves modulation of *Indy* transcription. Additionally, positive effects of altered *Indy* transcription have been demonstrated in mammals, where genetic manipulation to reduce *INDY* function imparts significant age-related health benefits on insulin signaling and metabolism that are also found in *Indy* long-lived mutant flies [7, 21]. The fact that *Indy* expression varies in the wild and imparts such significant improvements in fitness should not be understated. Both experimental and natural *Indy* genetic variants appear to act via influences acting on the *Hoppel* transposon. Numerous studies on metazoans suggest that domestication of transposons serves as a platform for the generation of useful genetic variants and novel gene regulatory networks, upon which natural selection may act [22-27]. In particular, recent data demonstrates that *Hoppel* double-stranded RNAs are generated *in vivo* from a trigger site called *Hoppel-killer(Hok)* on chromosome 4, and that this locus serves as a global regulator of gene silencing directed at *Hoppel* elements elsewhere in the genome [28]. Thus, the *Hoppel* insertion in *Indy* (along with other *Hoppel* elements throughout the genome) may well be under the influence of complex signals acting through small RNA pathways and heterochromatic gene silencing. The entry of the *Indy* gene into the *Hoppel* element global regulatory networks seems to have provided ample positive variation for natural selection to act upon. Further experiments will be necessary to delineate the precise mechanisms through which *Indy's* inclusion in this network generates the phenotypes we describe. Moreover, if evolution tinkers with *Indy* expression to regulate important life-history elements in Nature, it suggests that *Indy* may serve as a natural and important hub for small-molecule intervention linking metabolism, fitness and longevity.

## MATERIALS AND METHODS

### *Drosophila* Stocks

22 inbred *Drosophila* lines of different geographic origin were obtained from *Drosophila* Species Stock Center (https://stockcenter.ucsd.edu/). *Drosophila* Genetic Reference Panel (DGRP) stocks, DGRP lines, were original collected form Raleigh, NC, they were also known as RAL lines. The stock numbers of these lines are summarized in Table S1. All flies were maintained at 25°C in a temperature-controlled incubator at 50% humidity with a 12-hour light/dark cycle.

### PCR based Hoppel insertion phenotyping

Multiplex PCR using genomic DNA as template was performed with a combination of three primers: a common forward primer located upstream of *Hoppel* insertion (*Indy-hoppel*±1S: 5'-CTACATTGTATACGGAGACATTCG G-3'); two reverse primers, one inside *Hoppel* (*Indyhoppel*+3A: 5'-CCATGTAAATTCGTTTCTTCGATC-3') and the other downstream of *Hoppel* (*Indy-hoppel*−1A: 5'-CATCTTTCGTCTTGCTATCAGCA-3'). Homozygote for *Hoppel* insertion (+/+) leads to two amplicons: 392 *bp* (between *Indy-hoppel*±1S and *Indy-hoppel*+3A) and 1423 *bp* (between *Indy-hoppel*±1S and *Indy-hoppel*−1A). Homozygote for without-*Hoppel* insertion (−/−) can be identified by a single amplicon of 319 *bp* in size (between *Indy-hoppel*±1S and *Indy-hoppel*−1A). Short extension time (15 seconds) was used to eliminate the formation of large amplicon of 1423 *bp*. Therefore, +/+ homozygote can be visualized as a single amplicon of 392 *bp* and −/− homozygotes as a 319 *bp* amplicon. Heterozygote was identified as two amplicons, both the 392 *bp* and 319 *bp* ones.

### Isolation of +/+ and −/− homozygotes from Captain Cook, Oahu and Hidalgo lines

The crossing scheme for isolating both +/+ and −/− homozygotes from one inbred line is presented in Figure S1. Following this scheme, +/+ and −/− homozygous share the same ‘grandmother’ (generation *P*). The +/+ and −/− homozygotes, once isolated, were maintained via sib mating as stable stocks. We genotyped the homozygote stocks again at least 7 months after they had been generated and found their *Hoppel* insertion status unchanged. Therefore, the transposition rate of *Hoppel* is expected to be low and the homozygote lines can be considered as genetically stable.

### Quantification of *Hoppel* insertion allele frequency

The quantifications of insertion allele frequency were repeated twice with 28 months in between. In the first attempt, before +/+ and −/− homozygotes were isolated from *Cpt. Cook*, *Oahu* and *Hidalgo* lines, 10 adult flies were individually genotyped using the PCR assay described above and the allele frequency was calculated from the number of observed +/+, −/+ and −/− individuals. In the second attempt, performed after +/+ and −/− homozygotes were isolated, pooled genomic DNA from 50 individuals from each inbred lines were genotyped and the PCR product visualized on 1% agrose gels. The amplicon band density was measured with a ChemiDoc XRS camera system (BioRad) and the density ratios between the large and the small amplicon were used to calculate allele frequency through interpolation from a standard curve. The standard curve is established by measuring amplicon density ratio from a series of standard pooled genomic DNA samples of designed allele frequencies. The standard pooled DNA was prepared from 5 individuals and the expected frequencies in the samples ranged from 0.1 (one −/+ and four −/− individuals) to 0.9 (one −/+ and four +/+ individuals). The *Hoppel* insertion allele frequency in DGRP lines were also assessed using the pooled genomic DNA method, but with 5 individuals in each pooled sample. For the DGRP lines we assayed and reported in Figure 1C, we noticed all of them appear to be homozygous for either (+) or (−).

### Life span studies

Flies for demographic experiment were raised and collected from population density controlled broods in order to avoid any confounding effects due to over-crowding. The density controlled broods consisted of 10 replicate vials, each of which were seeded with 25 mating pairs of young adults as parents. The parents were allowed to mate and lay eggs for 2 days before being transferred onto fresh food. After 5 transfers, the parents were discarded. The offsprings from these parents were collected over a period of 24 hours and sorted by sex. 25 males and 25 females were kept in vials containing either 0.5N (5% yeast, 5% sucrose, 2% agar and 0.25% Tegospet in w/v concentration) or 1.5N (same as 0.5N except for 15% yeast and 15% sucrose) diet. For each genotype and dietary treatment, 10 replicate vials were set up so the total sample size is 250 for both sexes. Flies were transferred onto fresh food every two days with the dead flies, if any, removed and the number of death recorded. In the demographic assay for *Cpt. Cook*, *Oahu* and *Hidalgo* lines, heterozygote genotypes (*Hoppel* −/+) are generated by crossing −/− females to +/+ males. The inter-strain hybrid genotypes were generated from crosses between males and females from different lines, as summarized in Table S3.

### Fecundity assay for +/+, −/+ and −/− genotypes for Cpt. Cook, Oahu and Hidalgo lines

Flies for fecundity assay were collected from the density controlled broods set up the same way as for the demographic experiments. Offsprings from the density controlled broods were collect within 8 hours, to ensure only newly eclosed males and females were collected. Groups of 5 males and 5 females were then transferred into vials containing 0.5N diet. The flies were then transferred onto fresh food every day and the number of eggs produced during the previous day were counted and recorded. The average egg production per female was computed from the total eggs of replicate vials and the total number of females. Each genotype was assayed with at least 7 independent biological replicates.

### DNA sequencing of the region surrounding Hoppel element insertion in *Indy*

For +/+ and −/− homozygotes isolated from *Cpt. Cook*, *Oahu* and *Hidalgo* lines, DNA fragments both upstream and downstream of *Hoppel* element were amplified using the following primers: (*Hoppel*-BA: 5'-GAATTTCAGGTGTTTCGTGTGGG-3') and (*Hoppel*-BA-S: 5'-CAGATGTCTGCTATCAC TTTGTGCG-3') for upstream; (*Indy-hoppel*+1A: 5'-AAATCGTCTTCTAGCACAACACG-3') and (*Hoppel*-BS: 5'-GCATGAATAATGACCAGTTGAAC CC-3') for downstream. The PCR product was purified and sequenced using BigDye terminator chemistry on an Applied Biosystem platform. All fragments were sequenced on both strands. The sequence for DGRP lines was retrieved from DGRP Freeze 1 Data Release (http://www.hgsc.bcm.tmc.edu/projects/dgrp/freeze1_July_2010/). The coding sequence of *Indy* is highly conserved and there is only two nonsynonymous polymorphic sites (18822439A/G, resulting in an Ile/Val replacement change; and 18824503A/T, a Phe/Tyr replacement) with low frequency in all DGRP lines, 162 members in total. To confirm the observed effect of *Hoppel* insertion is not confounded by linked coding sequence polymorphisms in *Cpt. Cook*, *Oahu* and *Hidalgo* lines, we sequenced the coding regions for both +/+ and −/− homozygotes from the aforementioned lines. We found *Cpt. Cook* −/− homozygotes appeared to be fixed for 18822439G allele and all the other lines 18822439A allele. All of them are fixed for 18824503A. The 18822439A/G and 18824503A/T polymorphism locate at nucleotide position 3L: 18822439 and 18822503 respectively, based on *Drosophila* genome release version 5.

### *Indy* expression level quantification by qPCR method

Total RNA was extracted from 10-day old flies using TRIzol reagent (Invitrogen) and was further purified using RNA Miniprep columns (Qiagen). Total cDNA was reverse transcribed using iScript cDNA Syntesis kit (BIORAD). RT-qPCR assays were performed on an ABI 7500 Fast Real-Time PCR System thermocycler using SYBR Green PCR Master Mix (ABI) following standard protocols. The relative change in *Indy* expression level in reference to the level of GAPDH was computed using ddCT method provided by ABI 7500 Fast System Software. The qPCR primers for GAPDH detection were: GAPDH-F: 5'-GACGAAATCAAGGCTAAGGTCG-3'. GAPDH-R: 5'-AATGGGTGTCGCTGAAGAAGTC-3'. The qPCR primers for *Indy* transcript detection were: (*Indy*-A-9Q: 5'-TGTTTCAGTCCCTGGC −3') and (*Indy*-S-9Q: 5'-TGGGCGGAGTACTAACC −3'), located in exon 8 and exon 9 respectively. We sequenced *Indy* exon 8 and exon 9 and confirmed the primer binding sites are conserved and all our lines carry the identical sequence. The qPCR assays were all performed with 4 replicates.

## SUPPLEMENTAL INFORMATION


