# Supplementary material for: Indy gene variation in natural populations confers fitness advantage and life span extension through transposon insertion
Source: Aging (Albany NY). 2014 Jan 31;6(1):58–69. doi: 10.18632/aging.100634 (PMC3927810; doi:10.18632/aging.100634)
Supplement: Supplementary file 1 [file aging-06-058-s001.pdf]

SUPPLEMENTAL INFORMATION

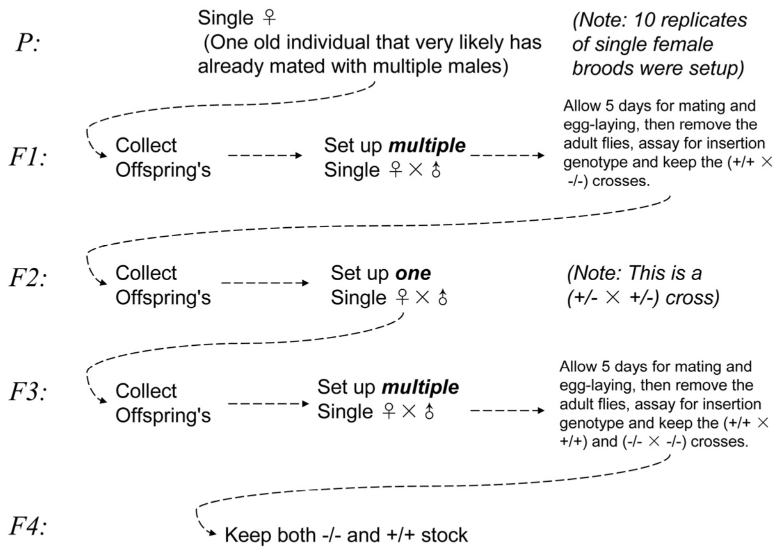

Supplementary Figure 1 related to Figures 1-3. Crossing scheme to obtain congenic heterozygotes (−/+) for Hoppel in Indy, homozygotes (+/+) for Hoppel in Indy and homozygotes without Hoppel in Indy (−/−).

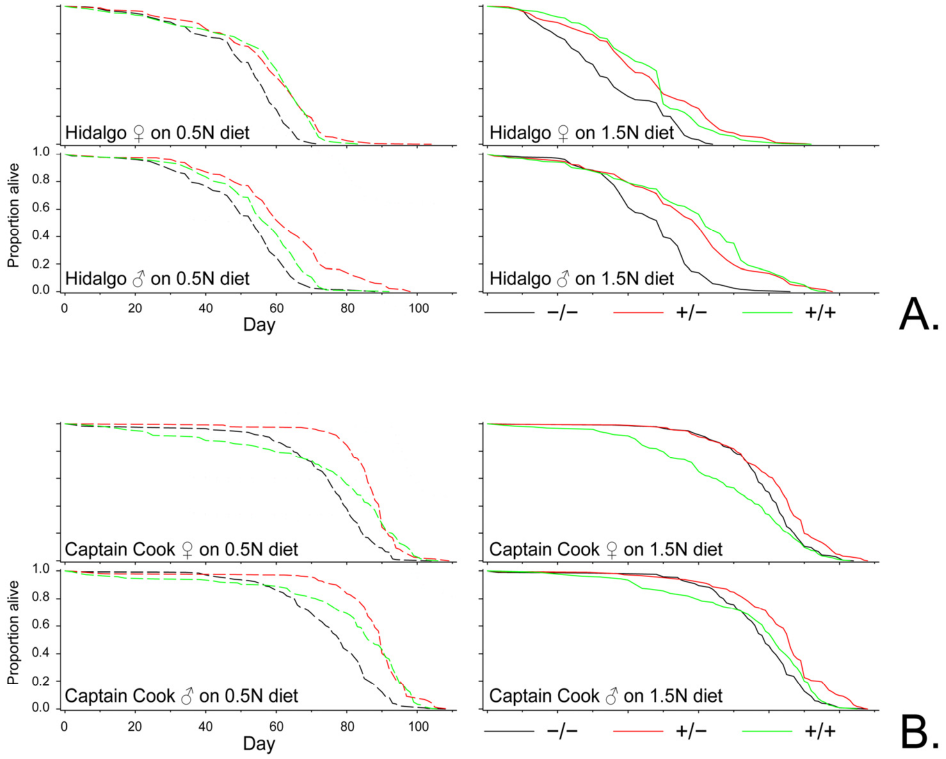

Supplementary Figure 2 related to Figure 2A and 2B. Survivorship plots for female and male *Hoppel* genotypes isolated from Hidalgo and Captain Cook lines with either no chromosome containing *Hoppel* in *Indy* (−/−; in black); one chromosome containing *Hoppel* in *Indy* (+/−; in red) or both chromosomes containing *Hoppel* in *Indy* (+/+; in green) grown on two different foods (1.5N and 0.5N diet) (*19*). Logrank and Boschloo tests are in Table S2.

Tables S1-S3

Supplementary Table S1 related to Figures 1-3. List of stocks:

Name	Stock center line No.	Source	Note
RAL208	25174	Bloomington Stock Center	DGRP lines, collected form Raleigh, North Carolina.
RAL313	25180		
RAL357	25184		
RAL358	25185		
RAL362	25187		
RAL365	25445		
RAL375	25188		
RAL379	25189		
RAL399	25192		
RAL427	25193		
RAL437	25194		
RAL517	25197		
RAL555	25198		
RAL639	25199		
RAL705	25744		
RAL712	25201		
RAL714	25745		
RAL732	25203		
RAL765	25204		
RAL774	25205		
RAL799	25207		
RAL820	25208		
RAL852	25209		
Monkey Hill	14021-0231.34	*Drosophila* Species Stock Center	Collected from St. Kitts Island, 2005
Mill Creek	14021-0231.55		Collected from Arkansas, 2007
Lujeri	14021-0231.76		Collected from Malawi, 2009
Athens	14021-0231.69		Collected from Greece, 1965
Israel	14021-0231.68		Collected from Haifa, Israel, 1954
Bahia	14021-0231.15		Collected from Brazil
Hidalgo-a	14021-0231.42		Collected from Mexico, 2005
Plainsville	14021-0231.56		Collected from Connecticut, 2007
Cpt Cook	14021-0231.54		Collected from Hawaii, 2007
Oahu	14021-0231.00		Collected from Hawaii, 1955
Catalina	14021-0231.47		Collected from Catalina Is., CA, 2006
Prescott	14021-2031.48		Collected from Arizona, 2006
Hidalgo-b	14021-0231.40		Collected from Mexico, 2005
Tucson	14021-0231.37		Collected from Arizona, 2005
Le Reduit	14021-0231.53		Collected from Mauritius, 2006
Ken-Ting	14021-0231.07		Collected from Taiwan, 1968
Mariana	14021-0231.05		Collected from Mariana Is., Guam, 1968
Kuala Lumpar	14021-0231.04		Collected from Malaysia, 1962
Ica	14021-0231.01		Collected from Peru, 1956
Koriba Dam	14021-0231.64		Collected from Zimbabwe, 1963
Cape Town	14021-0231.51		Collected from South Africa, 2007
Mumbai	14021-0231.45		Collected from India, 2006

Supplementary Table S2 related to Figures 2A and 2B. Statistical tests of the extension of survivorship and maximum lifespan. The *P* values for both the logrank test and the Boschloo test (*20*) are the probabilities associated with two-sided hypothesis.

Strain	Food	Gender	Genotypes		Lifespan extension of genotype #2 over #1 (days)			Logrank test for survivorship		Boschloo test for max. lifespan	
			#1	#2	Median	Mean	$Max_{(90\%)}$	*P*	χ^2	*P*	Boschloo
Cpt.Cook	1.5N	Female	+/+	+/−	12	13.31	4.5	2.15×10^{-11}	44.829	5.12×10^{-3}	4.59×10^{-3}
	1.5N	Female	+/+	−/−	8	10.49	0.9	5.13×10^{-4}	12.068	0.86049	0.86092
	1.5N	Female	−/−	+/−	4	2.82	3.6	6.21×10^{-5}	16.038	0.01526	0.01623
	1.5N	Male	+/+	+/−	4	7.01	2.7	2.00×10^{-5}	18.185	1.02×10^{-3}	2.56×10^{-5}
	1.5N	Male	−/−	+/+	3	1.53	2.7	0.12725	2.326	0.72493	0.78731
	1.5N	Male	−/−	+/−	7	5.47	0	1.21×10^{-8}	32.474	1.09×10^{-3}	1.05×10^{-4}
	0.5N	Female	+/+	+/−	5	10.93	1.8	0.06775	3.337	0.09592	0.10427
	0.5N	Female	−/−	+/+	6	1.19	2.7	1.02×10^{-8}	32.801	1.00×10^{-3}	9.77×10^{-7}
	0.5N	Female	−/−	+/−	11	12.13	4.5	$<1\times10^{-16}$	111.169	0.01465	0.02016
	0.5N	Male	+/+	+/−	3.5	6.73	2.7	0.06309	3.454	0.3375	0.3438
	0.5N	Male	−/−	+/+	8.5	5.30	1.8	1.37×10^{-12}	50.226	1.00×10^{-3}	1.39×10^{-6}
	0.5N	Male	−/−	+/−	12	12.03	0.9	$<1\times10^{-16}$	112.438	1.51×10^{-3}	6.38×10^{-4}
Oahu	1.5N	Female	+/+	+/−	1	2.55	2.7	0.78282	0.076	0.32401	0.37531
	1.5N	Female	−/−	+/+	16	10.67	13.5	2.76×10^{-10}	39.841	0.46357	0.4978
	1.5N	Female	−/−	+/−	17	13.22	10.8	4.08×10^{-13}	52.604	0.04994	0.0624
	1.5N	Male	+/+	+/−	16.5	16.06	20.7	$<1\times10^{-16}$	199.568	1.32×10^{-3}	4.77×10^{-4}
	1.5N	Male	−/−	+/+	2.5	0.18	25.2	0.01902	5.499	0.07516	0.12364
	1.5N	Male	−/−	+/−	19	16.25	4.5	2.09×10^{-11}	44.881	0.70869	0.71879
	0.5N	Female	+/+	+/−	7	7.71	3.6	2.13×10^{-10}	40.344	1	1
	0.5N	Female	−/−	+/+	2	5.49	0	0.37256	0.795	0.34869	0.37181
	0.5N	Female	−/−	+/−	9	13.20	3.6	4.01×10^{-10}	39.110	0.52204	0.56569
	0.5N	Male	+/+	+/−	16	16.06	17.1	$<1\times10^{-16}$	154.760	0.11213	0.12339
	0.5N	Male	−/−	+/+	5	1.23	11.7	0.42827	0.628	0.03751	0.0533
	0.5N	Male	−/−	+/−	21	17.30	5.4	$<1\times10^{-16}$	77.269	0.49926	0.62416
Hidalgo	1.5N	Female	+/−	+/+	4	0.13	0	0.40275	0.700	1	1
	1.5N	Female	−/−	+/+	18	10.54	25.2	2.07×10^{-12}	49.420	0.11253	0.12348
	1.5N	Female	−/−	+/−	14	10.41	25.2	6.31×10^{-14}	56.272	0.1081	0.12348
	1.5N	Male	+/−	+/+	2	2.19	1.8	0.13152	2.275	0.40261	0.41001
	1.5N	Male	−/−	+/+	12	12.33	9	$<1\times10^{-16}$	101.559	1.00×10^{-3}	7.03×10^{-6}
	1.5N	Male	−/−	+/−	10	10.14	10.8	1.11×10^{-16}	69.132	1.35×10^{-3}	6.78×10^{-4}
	0.5N	Female	+/−	+/+	2	0.79	18	0.43227	0.617	1	1
	0.5N	Female	−/−	+/+	8	5.81	10.8	1.11×10^{-16}	69.440	0.01076	0.01426
	0.5N	Female	−/−	+/−	6	6.60	28.8	3.33×10^{-15}	62.060	1	1
	0.5N	Male	+/+	+/−	4	6.31	5.4	3.97×10^{-9}	34.636	1.24×10^{-3}	3.31×10^{-4}
	0.5N	Male	−/−	+/+	4	4.59	3.6	1.85×10^{-5}	18.340	1	1
	0.5N	Male	−/−	+/−	8	10.91	9	$<1\times10^{-16}$	82.254	1.02×10^{-3}	3.31×10^{-5}

Supplementary Table S3 related to Figure 2C. Genotypes tested in inter-strain demography experiments.

Paternal \ Maternal	*Cpt.Cook* +/+	*Cpt.Cook* −/−	*Oahu* +/+	*Oahu* −/−	*Hidalgo* +/+	*Hidalgo* −/−
Cpt.Cook +/+	+/+ (*)	−/+ (*)	+/+ (†)	−/+ (†)	+/+ (†)	−/+ (†)
Cpt.Cook −/−		−/− (*)	−/+ (†)	−/− (†)	−/+ (†)	−/− (†)
Oahu +/+	+/+ (†)	−/+ (†)	+/+ (*)	−/+ (*)	+/+ (†)	−/+ (†)
Oahu −/−	−/+ (†)	−/− (†)		−/− (*)	−/+ (†)	−/− (†)
Hidalgo +/+	+/+ (†)	−/+ (†)	+/+ (†)	−/+ (†)	+/+ (*)	−/+ (*)
Hidalgo −/−	−/+ (†)	−/− (†)	−/+ (†)	−/− (†)		−/− (*)

(*): Crosses within lines
(†): Inter-strain crosses: crosses between different lines.
